# Minimally Invasive Thermal Ablation for Papillary Thyroid Microcarcinoma: Feasibility Analysis in Primary Healthcare Settings

**DOI:** 10.1155/ije/1475199

**Published:** 2025-10-10

**Authors:** Shaotian Li, Shanshan Hu, Xiaoli Zheng, Xiong Ku, Jingfeng Zou, Liping Wang, Guqiao Nie, Yiting Liu, Chunhui Tian, Jiajia Ran, Xin Yang, Mi Yan, Yilan Yin, Yun Liu, Jingjing Wan, Wen Peng

**Affiliations:** ^1^Union Hospital, Tongji Medical College, Huazhong University of Science and Technology, Wuhan, Hubei, China; ^2^Geriatric Hospital Affiliated to Wuhan University of Science and Technology, Wuhan, Hubei, China

**Keywords:** minimally invasive thermal ablation, papillary thyroid microcarcinoma, primary healthcare

## Abstract

Thyroid tumors are the most prevalent malignant neoplasms of the endocrine system, with an incidence approximately ten times higher than that of other endocrine tumors, accounting for 0.2%–1.0% of all malignant tumors (Zhu and Zhang, 2012 and Liu and Liao, 2017). Papillary thyroid microcarcinoma (PTMC) is a distinct subtype of papillary thyroid carcinoma (PTC), characterized by a maximum diameter of ≤ 10 mm. In recent decades, the incidence of PTC has tripled (Howlader, 2020). According to the 2014 World Health Organization (WHO) cancer report, over 50% of newly diagnosed thyroid cancer cases are PTMC (Soares et al., 2014). Furthermore, advancements in ultrasound (US) diagnostic techniques and the widespread adoption of US-guided fine-needle aspiration biopsy (US-FNAB) have led to a continued rise in the detection rate of PTMC (Bi, 2019). Among the various treatment modalities for PTMC, minimally invasive thermal ablation techniques offer substantial advantages over traditional surgical interventions, including enhanced safety, efficacy, minimal invasiveness, improved cosmetic outcomes, cost-effectiveness, ease of operation, and reduced anesthesia requirements. Moreover, these procedures are not confined to operating rooms and can be effectively performed in outpatient settings. Given these benefits, thermal ablation techniques hold great promise for widespread adoption in primary healthcare settings in China (primary healthcare facilities are critical for equitable access in resource-limited regions), as they not only reduce patient treatment costs but also optimize healthcare resource allocation, aligning with the national hierarchical medical system reform. This study aims to assess the feasibility of promoting the application of minimally invasive thermal ablation for PTMC in primary healthcare settings.

## 1. Introduction

Papillary thyroid microcarcinoma (PTMC), a distinct subtype of papillary thyroid carcinoma (PTC), is characterized by its small lesion size and relatively low risk of distant metastasis and local invasion [1–6]. In addition, PTMC is more commonly observed in younger patients [7]. While conventional open surgery remains the primary treatment modality, it presents several challenges, including surgical trauma, high costs, and a substantial risk of postoperative complications.

Minimally invasive thermal ablation, guided by ultrasound (US) for precise localization and controlled energy delivery, offers a promising alternative by achieving satisfactory therapeutic outcomes while reducing trauma, costs, and recovery time. This approach effectively addresses some limitations of traditional surgery. Furthermore, given its relatively simple and flexible operation, thermal ablation can be performed under local anesthesia in appropriately equipped outpatient settings. These advantages make it a valuable technique for broader implementation in primary healthcare facilities.

## 2. Current Status of PTMC Treatment

At present, conventional open surgery remains the primary treatment modality for PTMC, encompassing near-total thyroidectomy, total thyroidectomy, and unilateral lobectomy with isthmusectomy. While these surgical approaches have been proven effective, their drawbacks, such as high costs, surgical trauma, blood loss, prolonged postoperative recovery, and the incidence of complications, cannot be overlooked.

A study conducted by Kuma Hospital in Japan revealed that among 974 patients who underwent immediate surgery, the incidence of adverse events was significantly higher compared to 1179 patients who opted for active surveillance (AS) [8]. Among the complications associated with open thyroidectomy, hypocalcemia is the most common, with an incidence rate of 10%–30% [9], primarily due to parathyroid gland injury or compromised blood supply. In addition, the presence of a conspicuous cervical surgical scar and the necessity for thyroid hormone replacement therapy can severely impact patients' quality of life [10, 11]. Given these considerations, the potential drawbacks of open surgery may outweigh its benefits in some cases.

Internationally, AS is increasingly recommended as a management strategy for PTMC, also referred to as “delayed or deferred surgery.” Given that PTMC lesions measure ≤ 10 mm in diameter, exhibit slow progression, they rarely evolve into clinically significant thyroid cancer [12]. A study by Ito et al. in Japan followed 340 PTMC patients for up to 74 months [13] and found no significant differences in distant organ metastasis or lymph node metastasis between the AS group and the surgical resection group. These findings suggest that, due to its indolent biological nature, PTMC may be managed with AS in selected patients instead of immediate surgery. In fact, the 2015 American Thyroid Association (ATA) guidelines recommend AS as an alternative to traditional thyroidectomy [14].

Some PTMC patients remain asymptomatic throughout their lifetime, and even in cases with clinical symptoms or local lymph node metastases, overall survival rates remain largely unaffected [15], allowing for subsequent intervention if necessary. Therefore, AS is recommended as the first-line approach for patients with asymptomatic and nonmetastatic PTMC.

**Figure 1:**
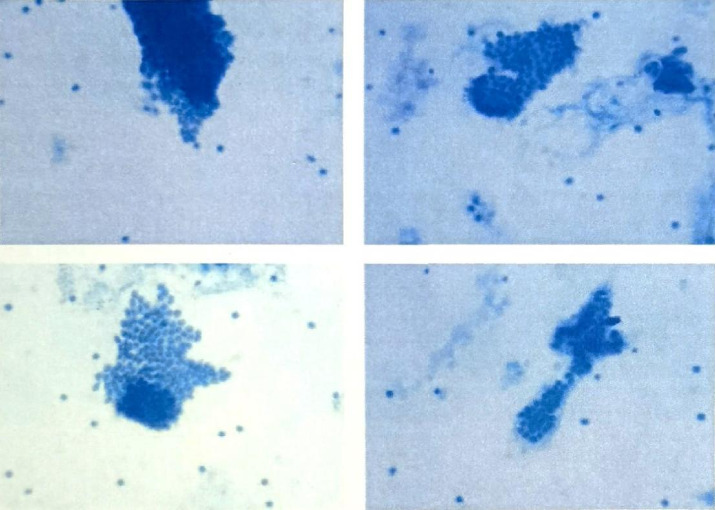
PTMC cytological smear.

However, AS has several limitations. First, although the prognosis of PTMC is generally favorable, certain cases may progress to more aggressive thyroid cancer or exhibit histological subtypes with high invasiveness, leading to local or distant metastases [16, 17], potentially worsening patient outcomes. Second, patients diagnosed with suspicious malignant nodules via US or fine-needle aspiration (FNA) biopsy (FNAB) but left untreated often experience significant anxiety and psychological distress, as the fear of living with cancer can negatively impact their quality of life [18, 19]. Most importantly, no definitive predictive marker (gold standard) has been established to accurately stratify PTMC patients by risk, making it challenging to determine which individuals are suitable for AS. Some researchers argue that AS should be regarded as a management option rather than a definitive treatment strategy, and further comparative studies between open surgery, nonsurgical treatments, and AS are still needed [20]. However, such studies remain limited (Figure 1) [21].

Bridging the gap between aggressive open surgery and conservative AS, a treatment modality has emerged that combines the advantages of both approaches: minimally invasive thermal ablation. This technique achieves a balance between cosmetic outcomes and therapeutic efficacy and includes microwave ablation (MWA), pulsed laser ablation (PLA), and radiofrequency ablation (RFA). Among these, RFA and MWA are more commonly used in China, whereas RFA and PLA are more frequently applied internationally.

## 3. Minimally Invasive Thermal Ablation Techniques

### 3.1. MWA

MWA utilizes a microwave antenna to deliver microwave energy directly into the target nodule, inducing rapid oscillation of water molecules within the tissue. This frictional motion generates heat, increasing the temperature to above 60°C, ultimately leading to thermal coagulation necrosis and effective lesion destruction. Under US guidance, a microwave needle is precisely inserted into the lesion, and the MWA system is activated. The entire ablation process is continuously monitored via US until the entire nodule is encompassed by a hyperechoic region. The procedure is considered complete when contrast-enhanced US confirms the absence of enhancement in the treated nodule [22]. MWA is widely used in China for the treatment of thyroid nodules and related disorders. Compared to RFA, MWA is less affected by electrical conductivity, tissue desiccation, and carbonization. Moreover, it is minimally influenced by blood flow perfusion, allowing for faster heating, shorter treatment duration, and a larger ablation zone. These characteristics make MWA particularly suitable for treating larger thyroid nodules (diameter > 2 cm) and other solid organ lesions such as liver and kidney nodules [23]. A study by Yue et al. [24] evaluated the efficacy of MWA in 222 patients with 477 thyroid nodules, with follow-up data available for 254 nodules. The mean nodule volume decreased from 2.13 ± 4.42 mL to 0.45 ± 0.90, with an average volume reduction of 65% ± 65%. Notably, 82.3% (209/254) of the nodules exhibited a volume reduction of more than 50%, and 30.7% (78/254) completely disappeared within 6 months postablation. The procedure demonstrated good tolerability, with only transient pain and temporary voice changes reported as adverse events, and no severe complications observed. Due to its high energy output and large ablation zone, MWA is particularly advantageous for larger lesions. However, for smaller lesions such as PTMC, although its efficacy is well-established, precise energy control is challenging. Inaccurate energy modulation may lead to excessive ablation of normal thyroid tissue or even unintended damage to adjacent structures, necessitating careful procedural optimization for PTMC treatment.

### 3.2. PLA

PLA is a thermal therapy that utilizes laser energy to irradiate tissues, inducing a localized heating effect. In the treatment of thyroid nodules, PLA involves the insertion of a laser fiber to deliver energy directly to the lesion. Upon absorption of laser energy, the temperature of the targeted tissue rises to 60°C–100°C, leading to thermal coagulation necrosis. Under US guidance, a coaxial needle is inserted along the long axis of the thyroid nodule to the target site. After removing the inner stylet, a laser fiber is advanced to the same location, with its tip extending 5 mm beyond the needle tip while maintaining a minimum distance of 15 mm from the thyroid capsule and adjacent cervical structures, and hydrodissection is necessary to protect these important anatomical structures. Laser emission is activated, and the ablation process is continued until the hyperechoic vaporization zone at the fiber tip fully encompasses and extends beyond the lesion, at which point the procedure is terminated. PLA is characterized by a fine puncture needle, high maneuverability, and precise localization. During the ablation process, real-time US imaging allows for clear visualization of the echogenic changes at the fiber tip, ensuring accurate assessment of the ablation zone. These features make PLA particularly suitable for treating small nodules (diameter < 1 cm) or lesions adjacent to critical anatomical structures [25]. However, the high cost of laser equipment and its suboptimal efficacy in treating cystic, colloid, or large-volume nodules limit its widespread clinical application. In addition, there is a lack of research data on PLA treatment for PTMC, especially in comparison to RFA and MWA treatments for PTMC, which may become a hot topic for future research. Further advancements and refinements are necessary to optimize its role in thyroid nodule management.

### 3.3. RFA

**Figure 2:**
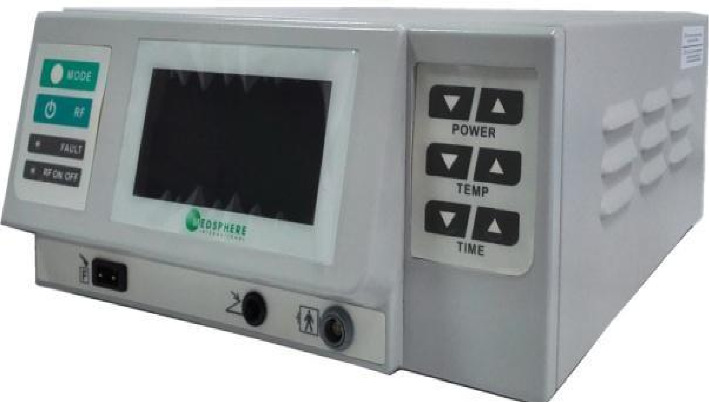
Radiofrequency ablation therapeutic instrument.

**Figure 3:**
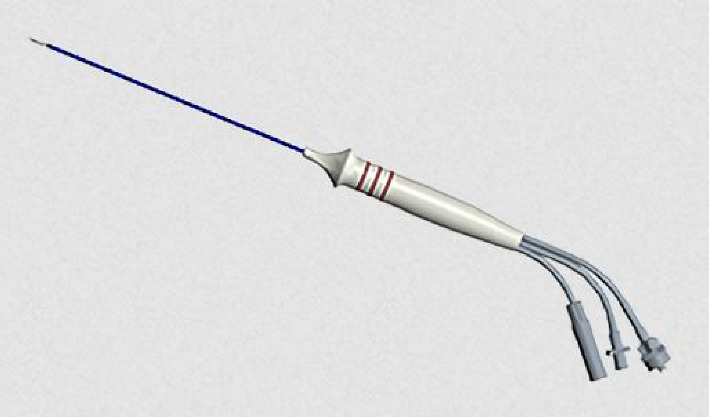
Radiofrequency ablation electrode needle.

RFA is a thermal ablation technique [26]. Its mechanism involves the generation of high-frequency alternating current by a radiofrequency generator, which is delivered to the target tissue through an electrode needle. This induces oscillation and friction among polarized molecules and ions within the surrounding tissue, converting electrical energy into thermal energy (Figures 2 and 3) [21]. The resulting localized hyperthermia causes irreversible coagulative necrosis of cellular proteins, leading to tissue destruction and subsequent resorption by the body. Ultimately, RFA inactivates soft tissue lesions and cancerous foci. Compared to MWA and PLA, RFA demonstrates a superior posttreatment volume reduction rate. Two meta-analyses on thermal ablation for benign thyroid nodules found that RFA achieved greater volume reduction than PLA (RFA: 92.2% and 87%; PLA: 43.3% and 44%) and had a lower overall complication rate (RFA: 1.3%; PLA: 1.8%) [27, 28]. In addition, a 5-year multicenter study by Bernardi et al. [29] reported a lower recurrence rate for nodules treated with RFA (20% vs. 38% for PLA). Patients undergoing PLA were more likely to require repeat treatment compared to those treated with RFA. Yan et al. [30] compared the efficacy of RFA and MWA in treating benign thyroid nodules, showing that RFA resulted in a greater volume reduction at both 6- and 12-month follow-ups. A meta-analysis comparing the efficacy and complications of RFA, MWA, and PLA in PTMC treatment revealed that their average volume reduction rates were 99.3% (RFA), 95.3% (MWA), and 88.6% (PLA), while their overall complication rates were 1.7% (RFA), 6.0% (MWA), and 0.92% (PLA) [31]. Among the three techniques, RFA delivers moderate energy output, falling between MWA (higher energy) and PLA (lower energy). This makes RFA particularly suitable for treating small-to-medium lesions (1-2 cm in diameter). Given that PTMC is defined by a maximum diameter of ≤ 1 cm, the ablative margin typically extends 2-3 mm beyond the lesion to ensure complete ablation. RFA's ability to precisely cover this margin minimizes excessive ablation while effectively eliminating the tumor, thereby reducing damage to surrounding normal tissues [32].

## 4. Application of Minimally Invasive Thermal Ablation Technology in Primary Care Facilities

**Figure 4:**
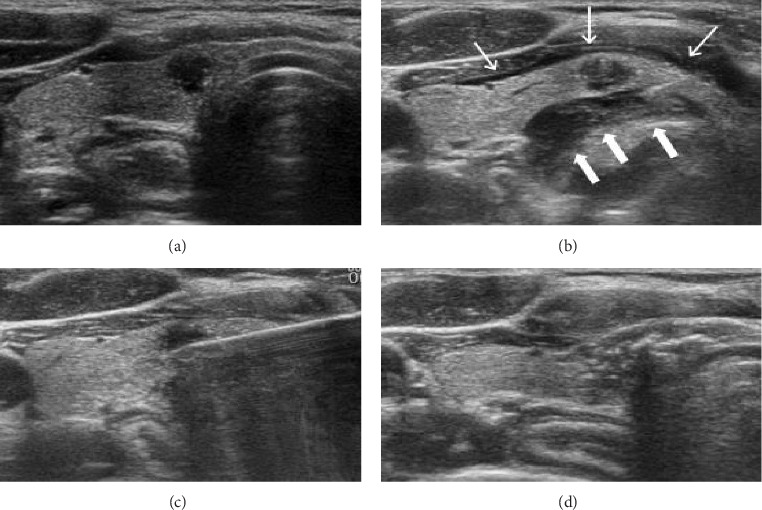
An example of the RFA procedure. (a) PTMC was confirmed by US-FNAB, located in the right lobe, near the trachea. (b) The administration of local anesthesia with 0.5% lidocaine (thin arrow) injected at the puncture site and around the thyroid capsule. Due to the lesion's proximity to the trachea, 10 mL of normal saline was injected for water isolation (thick arrow). (c) The radiofrequency ablation electrode was slowly placed from the puncture point to initiate ablation. (d) The changes in the echo of the lesion postablation, with the ablation area expanding to include both the primary lesion and normal thyroid tissue.

Under the leadership of Professor Song Mu at the Department of Thyroid and Breast Surgery, the Seventh Affiliated Hospital of Southern Medical University provides free thyroid and breast screening services to residents of Nanhai District, Foshan, Guangdong Province. The department identifies cases in the community, provides effective treatment plans on-site, and admits patients suitable for thermal ablation therapy for inpatient treatment. During this period, a randomized clinical controlled trial was conducted comparing RFA with traditional open surgery for treating PTMC [21]. The study compared the efficacy, complications, pain assessment, surgical metrics (operation time, hospitalization duration, intraoperative blood loss, and hospitalization costs), and postoperative quality of life between the two methods. After 1 year of follow-up, neither group experienced recurrence or distant metastasis, and the therapeutic outcomes were found to be comparable (Figure 4) [21]. Regarding complications, the RFA group had one patient, who complained of neck distension and a foreign body sensation in the throat (6.7%), as well as mild hoarseness (3.3%), all of which resolved rapidly. In contrast, over 50% of patients in the surgical group reported discomfort, including hoarseness (30%), dysphagia and aspiration (16.7%), and hand and foot twitching (6.7%), which took longer to resolve. In addition, one patient in the surgical group developed neck swelling on the night following surgery (3.3%), accompanied by difficulty in breathing and worsening symptoms, leading to an emergency procedure for thyroid region exploration, hemostasis, and hematoma removal. Postoperatively, the patient's breathing difficulty improved, but symptoms such as dry coughing and tracheal irritation persisted. These cases highlight that traditional surgery not only carries numerous complications but also poses life-threatening risks, such as hematomas. Regarding postoperative pain, the RFA group primarily experienced burning pain due to thermal effects, which gradually subsided after the ablation. As assessed by the visual analog scale (VAS), most patients in the RFA group reported mild pain, with no moderate or severe pain, and complete pain relief within 1 week. Some patients reported no pain after the procedure. In contrast, the surgical group had visible neck incisions, and open surgery inevitably caused damage to surrounding tissues, leading to more intense and prolonged postoperative pain. Some patients reported neck discomfort lasting 1 month or more postsurgery. Furthermore, the pain management in the surgical group required higher doses and a greater variety of analgesic medications compared to the RFA group, further indicating that open surgery resulted in more pronounced postoperative pain. In addition, the RFA group had significantly shorter operation times, shorter hospitalization periods, less intraoperative blood loss, and lower hospitalization costs compared to the surgical group. In terms of quality of life, the RFA group outperformed the traditional surgical group [21].

In summary, RFA is a safe, reliable, and cost-effective treatment option, alongside other minimally invasive thermal ablation techniques such as MWA and PLA. However, minimally invasive thermal ablation is not without limitations. Compared to traditional surgery, thermal ablation may result in incomplete ablation of the lesion, potentially increasing the risk of recurrence or metastasis over time. Furthermore, for lesions with lymph node invasion, minimally invasive ablation cannot achieve lymph node dissection, and surgical resection with lymph node removal remains necessary in such cases [33, 34]. In addition, since the follow-up period is relatively short, long-term differences in recurrence or metastasis rates between the two methods cannot yet be assessed, necessitating further studies and observation for conclusive results.

## 5. Feasibility Analysis of Minimally Invasive Thermal Ablation Technology in Primary Care Facilities

With the widespread use of high-resolution US diagnostic technology and FNA biopsy, the detection rate of thyroid malignancies has steadily increased, particularly for PTMC, which shows the most significant rise. Patient visits often reveal that most individuals discover thyroid nodules during physical exams or screenings, where a thyroid US is conducted. Based on imaging evaluations suggesting malignancy, physicians recommend FNA to confirm the diagnosis, ultimately identifying the nodule as malignant (e.g., PTC or PTMC). These patients typically do not seek healthcare due to local or systemic symptoms, but rather through routine screening. Therefore, PTMC is considered a silent disease, without obvious symptoms. Hence, early detection, diagnosis, and treatment of PTMC are essential. In contrast to the traumatic and complex nature of open surgery, minimally invasive thermal ablation technologies offer several advantages: they are cost-effective, efficient, minimally invasive, and aesthetically favorable, with lower anesthesia requirements. The procedure is relatively simple, and experienced physicians can perform it outside the operating room in a well-equipped outpatient setting, making treatment more accessible and conserving substantial healthcare resources. This also underscores the feasibility of applying these technologies in primary care facilities.

We propose establishing specialized outpatient clinics and treatment units in primary care facilities, integrating thermal ablation technologies into community healthcare. This model could combine screening, diagnosis, and follow-up care in a cohesive intervention system. Regular thyroid disease screenings could be conducted in community populations, allowing for early detection of cases. After evaluation by specialists, patients highly suspected of PTMC can be referred to outpatient clinics in grassroots institutions for further pathological confirmation and concurrent treatment. Leveraging the management and resource advantages of grassroots and community healthcare, long-term postoperative follow-up and health management for PTMC patients can be implemented, enabling full-cycle management of the disease. In addition, many benign thyroid conditions, such as nodular goiter, inflammatory nodules, thyroid cysts, and even hyperthyroidism [35], can also be treated with minimally invasive ablation in grassroots healthcare facilities. This approach not only improves patient convenience and reduces treatment costs but also alleviates the burden on tertiary hospital operating rooms, helping conserve valuable surgical resources.

In conclusion, promoting minimally invasive thermal ablation technologies in primary care facilities is both feasible and beneficial, offering an accessible, cost-effective, and resource-efficient solution for PTMC and other thyroid-related conditions.

## 6. Challenges and Prospects

Despite the clear advantages of minimally invasive thermal ablation technologies, significant challenges remain in introducing this technology into primary care facilities and establishing a mature intervention model. First, the high cost of ablation equipment may render it unaffordable for smaller grassroots institutions. Second, physicians performing these procedures must undergo long-term, systematic training and gain substantial clinical experience to master standard operating procedures and effectively manage emergency situations. Currently, primary care facilities lack such specialized professionals. Lastly, public awareness of minimally invasive thermal ablation remains relatively low, and patient compliance is often poor, making it difficult to implement large-scale screening and long-term follow-up management within community populations. However, the author suggests that under the framework of China's tiered medical system, a positive interaction and collaboration between large tertiary hospitals and primary care facilities will gradually emerge. This collaboration would not only facilitate patient referrals but also encourage the exchange of talent and technology. For example, necessary equipment could be introduced into appropriately scaled grassroots institutions, and specialists from tertiary hospitals could provide technical guidance and training. This approach would help cultivate competent operators capable of independently performing the procedures, thereby alleviating the workload of higher-level hospitals, conserving surgical resources, and fostering a positive bidirectional interaction. In addition, further large-sample, multicenter clinical studies are essential to verify the therapeutic effects of minimally invasive thermal ablation technologies across different patient populations. These studies would provide more reliable scientific evidence for the broader application of this technology in grassroots healthcare. Finally, strengthening public education and awareness about minimally invasive ablation technologies and thyroid diseases is essential to enhance patient recognition and compliance.

As a novel approach to treating thyroid diseases, minimally invasive thermal ablation technology holds significant potential in grassroots healthcare settings. Through further research and promotion, it is expected to offer safer and more effective treatment options for primary care services, ultimately improving patient care and overall healthcare experiences.

## Data Availability

The data results in this study are publicly available and can be used.
